# Associated factors for cognitive impairment in the rural highly elderly

**DOI:** 10.1002/brb3.1203

**Published:** 2019-04-01

**Authors:** Hyeyun Kim, Sujin Lee, Bon D. Ku, Su Geun Ham, Woong‐Sub Park

**Affiliations:** ^1^ Department of Neurology Catholic Kwandong University College of Medicine, International St. Mary’s Hospital Incheon Korea; ^2^ Sokcho Health Center Sokcho Korea; ^3^ Department of Preventive Medicine & Public Health Catholic Kwandong University College of Medicine Gangneung Korea

**Keywords:** cognitive impairment, dementia, rural health

## Abstract

**Background:**

Among the symptoms commonly faced by an increasingly aged population, dementia, preceded by cognitive impairment, most threatens their quality of life. Dementia is a well‐recognized burden, not only for individuals who face the disease and for their families, but also for entire nation.

**Aims:**

The purpose of this study was to identify associated factors for cognitive impairment in a very elderly population via a screening study design in Sokcho, a rural area in South Korea.

**Methods:**

Trained nurses screened patients from 75–84 years of age for dementia using the Mini‐Mental State Examination for Dementia Screening (MMSE‐DS) and conducted interviews to determine their socioeconomic status, education level, and living conditions (i.e., with or without family).

**Results:**

In total, 4,369 subjects (1,646 males, 2,723 females) were enrolled in this study. Reported MMSE‐DS scores decreased with increasing age and to a greater degree in less educated subjects (*p* < 0.001). Subjects requiring medical assistance or living alone exhibited lower MMSE‐DS scores compared to those who did not require medical assistance or who lived alone (*p* < 0.001).

**Conclusion:**

We found that less education, lower socioeconomic status, and living alone were associated factors for cognitive impairment based on this study of an elderly population.

## 1 INTRODUCTION

South Korea is known for one of the most rapidly aging society in the world. The elderly dependency ratio increased from 6.1% in 1980 to 15.2% in 2010 and is expected to be 20.0% in 2018, categorizing South Korea as an aged society (Bruti et al., 2016). In most of advanced society, a heavier burden of socioeconomic cost to the elderly person has been issued and one of the largest burdens is the cost pertaining to the healthcare system. In Korea, the burden of dementia was 5,117 per 100,000 those over the age of 65 years in year 2010 (Park, Eum, Bold, & Cheong, 2013). Dementia is a common neurodegenerative disease among elderly people. In South Korea, the prevalence of dementia has been increased during past years, especially in rural area (Suh, Kim, & Cho, 2003). Dementia requires more socioeconomic costs than any disease in the elderly, as it requires greater economic cost to care for the patients as well as medical treatment. However, the health care policies to the dementia have not kept up with the aging population in South Korea. Especially in rural area, many disease are diagnosed late due to poor access to medical service. In particular, cognitive decline in the highly elderly is not recognized as a disease, dementia. The elderlies and their family tend to recognize the decline of cognitive function as a symptom of natural aging. This is why it is common for rural demented elderly people to be neglected without proper medical intervention (Woo et al., 1998). To prevent this problem, the prevalence monitoring of specific disease such as cognitive decline is essential to plan and establish the health policy (Green & Zhang, 2016). We investigated the prevalence and associated factors for cognitive decline of the population aged 75–84 years that is at a high risk for cognitive decline in a rural area in South Korea through complete enumeration survey.

## 2 METHOD

Sokcho is located at approximately 200 km apart from Seoul to the east. It is a fishing hamlet near the Sea of Japan (East Sea). The extent of Sokcho is approximately 105.30 km^2^ and the population is 84,180, with 12,261 individuals aged ≥65 years, as for the survey conducted in 2014. As per the recent OECD data (https://data.oecd.org/pop/elderly-population.htm), Japan has nearly 12.7% of elderly aged ≥65 years, which probably is not much different from the 14.9% in rural setup. Sokcho is already an aged society at the time of survey. This survey conducted all 4,624 subjects aged 75–84 years living in Sokcho. The subjects aged 75–84 years living in Sokcho performed the Korean version of Mini‐Mental State Examination for Dementia Screening (MMSE‐DS) by a trained nurse staff (Kim et al., 2010) and were interviewed by a trained nurse staff and two field (social) workers. MMSE‐DS includes several domain functions such as orientation, attention, calculation, memory language, and visuo‐spatial ability using a structured, 30‐point questionnaire. A cut‐off point of 24 was used to consider cognitive impairment in several previous studies, which revealed a sensitivity of 85% and specificity of 90% (Creavin et al., 2016).

The survey was performed in accordance with the Declaration of Helsinki guidelines and written informed consent was obtained from all participants. We conducted this study after IRB approval (IS16EASI0024). Field workers obtained informed consent from all the subjects after explaining the process and the aim of this survey. Upon agreement by subjects, they were asked to sign or provide thumb impressions on the consent form. The information included the duration of education, living alone or family, the status of national medical insurance or medicare services, and medical history.

We evaluated the demographics from the screening data. We divided the subjects into two groups: healthy elderly and cognitive impairment based on the MMSE‐DS score. Cognitive impairment was defined as <24 points on MMSE‐DS. The two groups were analyzed for independent associated factors: age, duration of education, living conditions, and national health insurance type. The duration of education was conducted through interview with the subjects or family members and divided into four groups; 0–3 years, 4–6 years, 7–12 years, and above 13 years depending on the past years of public education. The living condition is defined as whether the subject lives alone or with the family members. The Korean health insurance system covers the whole population residing within the territory of Korea. The main funding consist of insured persons and government grants. Economically difficult household get help with medical support from the government. The Korean health insurance could be reflective of the household economic status. For this reason, we analyzed the insurance type as economic status in this population. The data are expressed as numbers and percentages for a categorical variable or the mean and standard deviation for continuous variables. Student’s *t *test was used to examine the difference of means, and χ^2^ test was used examine that of percentages. Statistical analysis was performed using the SAS software for Windows, Version 9.4 with a *p *value <.05 considered statistically significant.

## 3 RESULT

The total number of subjects aged 75–84 years old living in Sokcho, South Korea was 4,624. The neurocognitive evaluation test was conducted for 3 years from 2014 to 2017. During that period, 53 elderly people died and 31 subjects moved to another area. Seventy‐six elderly people were excluded from the evaluation test because of difficulty in finding their place of residence and poor health condition. In addition, 95 people were not able to conduct the evaluation because the subject or family refused to participate in this study. Complete data was available for 4,369 subjects (94.4%, male; 1,646, female; 2,723).

The basic demographic characteristics are given in Table 1. The MMSE‐DS score was higher in men (25.4 ± 4.5) than that in women (22.8 ± 5.1) (*p* < 0.01). Overall, 47.5% of men had been educated for 7–12 years, and about half (51.2%) the women had been educated for 3 years or less. The rate of highly educated persons with an education duration of >7 years was higher among males (12.2%) than that among females (1.1%) (*p* < 0.01). The overall education duration was significantly longer in among males. The proportion of subjects living alone was higher among women (38.4%) than that among men (17.1%) (*p* < 0.01). The subjects supported by medical assistance were 14.3% and 17.6% among men and women, respectively (*p* = 0.0041).

**Table 1:** Characteristics of study population at baseline (*n* = 4,369)

Variables	Total (*n* = 4,369)	Men (*n* = 1,646)	Women (*n* = 2,723)	*p* Value
MMSE (mean ± *SD*)		25.4 ± 4.5	22.8 ± 5.1	<0.001
Age (year)				
75–77	1,765 (40.4)	710 (43.1)	1,055 (38.7)	<0.001
78–80	1,413 (32.3)	567 (34.5)	846 (31.1)	
81–84	1,191 (27.3)	369 (22.4)	822 (30.2)	
Education (year)				
0–3	1,636 (37.5)	242 (14.7)	1,394 (51.2)	<0.001
4–6	1,253 (28.7)	421 (25.6)	832 (30.6)	
7–12	1,245 (28.5)	781 (47.5)	464 (17.1)	
≥13	232 (5.3)	201 (12.2)	31 (1.1)	
Living type				
Living with family	3,042 (69.6)	1,365 (82.9)	1,677 (61.6)	<0.001
Living alone	1,327 (30.4)	281 (17.1)	1,046 (38.4)	
Nation health insurance				
Health insurance	3,647 (83.6)	1,406 (85.7)	2,241 (82.4)	0.0041
Medical assistance	714 (16.4)	234 (14.3)	480 (17.6)	

Note

MMSE: Mini‐Mental State Examination.

**Figure 1:**
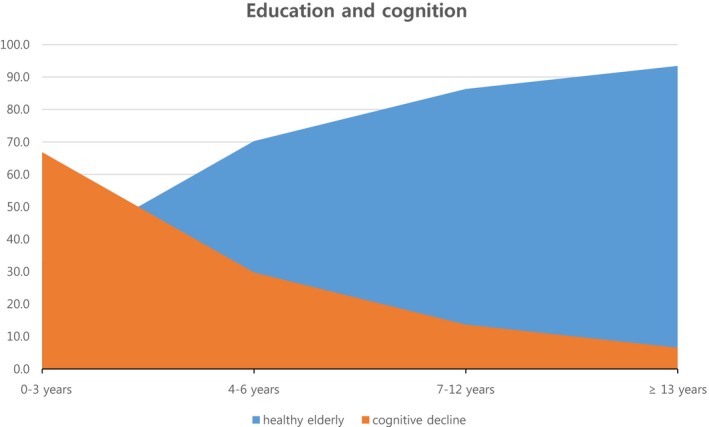
Education and cognition. The more educated group showed decreased cognitive impairment. Short duration of education (0–3 years) group showed higher proportion of cognitive impairment

Based on MMSE‐DS, the subjects were divided into two groups: cognitive impairment (*n* = 1,590) and healthy elderly (*n* = 2,779). The associated factors for cognitive impairment are given in Table 2. The mean MMSE‐DS score was 18.7 ± 4.9 in the cognitive impairment group and 26.7 ± 1.7 in the healthy group (*p* < 0.01). Based on age, the group aged 81–84 years showed increased cognitive impairment at 52.9% (*p* < 0.01). The group aged 75–77 years comprised 71.1% of the healthy elderly. The duration of education was divided into four groups (0–3 years, 4–6 years, 7–12 years, and above 13 years). The shortest duration group (0–3 years) showed the highest proportion (66.8%) of cognitive impairment (Table 2). Highly educated subjects showed the highest MMSE‐DS scores (*p* < 0.01). The slope at 4–6 years of education was steep compared with that at other education groups (Figure 1). Cognitive impairment was more common among subjects living alone compared with that among subjects living with family members (*p* < 0.01). The proportion of cognitive impairment in the medical assistance group (45.5%) was higher than that in the health insurance group (34.7%) (*p* < 0.01).

**Table 2:** Risk factors for cognitive impairment

Variables	Healthy elderly (*n* = 2,779)	Cognitive impairment (*n* = 1,590)	*p* Value
	*N *(%)	*N *(%)	
MMSE‐DS (mean ± *SD*)	18.7 ± 4.9	26.7 ± 1.7	<0.001
Gender			
Male	1,334 (81.0)	312 (19.0)	<0.001
Female	1,445 (53.1)	1,278 (46.9)	
Age (year)			
75–77	1,279 (72.5)	486 (27.5)	<0.001
78–80	939 (66.5)	474 (33.5)	
81–84	561 (47.1)	630 (52.9)	
Education (year)			
0–3	580 (35.5)	1,056 (64.5)	<0.001
4–6	890 (71)	363 (29)	
7–12	1,089 (87.5)	156 (12.5)	
≥13	218 (94)	14 (6)	
Living type			
Living with family	1,995 (65.6)	1,047 (34.4)	<0.001
Living alone	784 (59.1)	543 (40.9)	
Nation health insurance			
Health insurance	2,383 (65.3)	1,264 (34.7)	<0.001
Medical assistance	389 (54.5)	325 (45.5)	

Note

MMSE: Mini‐Mental State Examination.

The MMSE‐DS score is shown in Table 3. The score (25.3 ± 4.6) in female was higher than that of male (23.6 ± 5.1) (p < 0.01). With increasing the duration of education, the score of MMSE‐DS showed also increased (*p* < 0.01). Subjects supported with national insurance service/medical assistance service accounted for 23.8 ± 5.0/22.5 ± 5.8 score of MMSE‐DS, respectively (*p* < 0.01).

**Table 3:** The MMSE‐DS score

Variables	MMSE‐DS score		*p* Value
	Mean	*SD*	
Gender			
Male (*n*)	23.6	5.1	<0.001
Female (*n*)	25.3	4.6	
Age (year)			
75–77	24.8	4.2	<0.001
78–80	24.2	4.6	
81–84	21.9	6.1	
Education (year)			
0–3	21.2	5.1	<0.001
4–6	24.4	4.7	
7–12	26.0	4.0	
≥13	27.1	3.0	
Living type			
Living with family	23.8	5.1	<0.001
Living alone	23.2	5.2	
Nation health insurance			
Health insurance	23.8	5.0	<0.001
Medical assistance	22.5	5.8	

Note

MMSE‐DS: Mini‐Mental State Examination for Dementia Screening.

The age‐dependent MMSE‐DS score is shown in Table 4. Fast score decline of MMSE‐DS was done at 81 ‐ 82 years old. However, in the male group, this fast decreasing of MMSE‐DS did not showed. The score of MMSE‐DS decreased stepwise according to aging.

**Table 4:** Age‐related MMSE‐DS score among elderly men and women in rural Korea

Variables	Total			Male			Female		
Age (*n*)	Mean	*SD*	*p *Value	Mean	*SD*	*p *Value	Mean	*SD*	*p *Value
75	25.0	4.2	<0.001	26.0	3.6	<0.001	24.2	4.5	<0.001
76	24.7	4.3		25.7	4.1		24.0	4.3	
77	24.7	4.0		26.1	3.6		23.9	4.0	
78	25.0	3.8		26.8	2.9		23.7	3.9	
79	24.2	4.8		25.2	4.9		23.5	4.5	
80	23.1	5.2		24.8	4.5		22.1	5.3	
81	23.1	5.5		24.7	5.1		22.3	5.5	
82	21.9	6.1		24.2	6.0		20.7	5.9	
83	21.1	6.3		23.6	4.4		20.0	6.7	
84	21.0	6.3		22.8	7.9		20.5	5.7	

Note

MMSE‐DS: Mini‐Mental State Examination for Dementia Screening.

## 4 DISCUSSION

The epidemiology of dementia has provided inconsistent results based on diagnostic criteria, the population age, and study assessments used. Age‐standardized prevalence for those aged ≥65 years was 8.2%–10.4% (Bruti et al., 2016; Kim et al., 2014). The age‐related prevalence of dementia showed 18%–20% for the elderly aged >75 years and 35%–40% for those aged >85 years (Kim et al., 2014). The prevalence showed a sharp increase with aging, particularly for those aged >75 years (Ohara et al., 2017; Wimo et al., 2017; Wimo, Winblad, & Jonsson, 2010). Understanding the characteristics of the age group with a high prevalence is needed to establish policies for preventing dementia and cognitive impairment. This study targeted the age group of 75–84 years. Our results were consistent with those of previous reports (Kim et al., 2014). The subjects aged 81–84 years showed cognitive impairment. The incidence was sharply increased in subjects aged 80 years.

The results pertaining to associated factors such as age, sex, and education were consistent with general findings in literatures (Chen et al., 2017; Lee, Liang, Peng, Chiou, & Chen, 2017; Olayinka & Mbuyi, 2014). Education is a well‐known protective factor against cognitive impairment and dementia (Chen et al., 2017; Tolppanen et al., 2014). Education could make the cognitive reserve that accumulates during their lifelong, therefore aiding the protection of cognitive impairment (Ihle et al., 2017). In the meta‐analysis with 15 prospective cohort studies, it was revealed that dementia risk was reduced by 7 % for per year through increased period of education (Xu et al., 2016). According to this equation, 6 years of education could reduce the risk of dementia by about 42% compared to those who have never received education. In this study, 53.5% of cognitive impairment showed a difference between 0 and 3 years of education and 4 and 6 years of education. Although this is a simple calculation, it seems to be a result that is not far different from the results obtained with meta‐analysis.

In our study, 6 years of education was enough to offer protection. Less than 6 years of education among elderly showed a higher prevalence of cognitive impairment compared with more than 6 years of education. We observed reduced cognitive impairment with an increased duration of education. Therefore, a longer period of education could provide a stronger protective effect for dementia. A 3‐year increase from 6 to 9 years decreased the prevalence of cognitive impairment from 32.9% to 15.5% in this study. Three educational years could cause the butterfly effect on maintaining of cognitive function, 60 years after. Elderly welfare, social economic burden, and academic training should be considered when establishing educational policies.

Single study of the relationship loneliness and dementia, the elderly who felt lonely had higher prevalence of dementia (Rafnsson, Orrell, d'Orsi, Hogervorst, & Steptoe, 2017). However, living alone among elderly population was not previously found to be an associated factor for dementia. The population of the elderly who live alone increased with social aging. The government of an aging society needs to establish appropriate policies to support senior citizens who live alone. There was a sex‐based difference. For elderly women, feeding and housing themselves was easier than that for men. In addition, the life span of women was longer than that of men (Strombach, Margittai, Gorczyca, & Kalenscher, 2016). Because of these reasons, the incidence of elderly women living alone was more common than that of elderly men living alone (Noh, Kim, Park, Hong, & Kwon, 2017). The elderly with a more severe declined cognitive function cannot live alone and need help for their activities of daily living (Margolis & Verdery, 2017). In this study, the incidence of cognitive impairment was higher in the living alone group than that in the living with family group. The correlation between living alone and cognitive impairment was not clear due to the cross‐sectional cohort study design. In our literature review, living alone has not been reported as an associated factor for dementia and cognitive impairment. The elderly who live alone have fewer chances to communicate with others (Margolis & Verdery, 2017). It could make the person feel lonely and depressed (Stahl, Beach, Musa, & Schulz, 2017). This would have a negative effect on maintaining cognitive function in the elderly living alone. In addition, the rural residence setting could have remoteness due to the low population density. It could aggravate withdrawal from community (Barth, Nickel, & Kolominsky‐Rabas, 2018).

For preventing cognitive impairment in the highly elderly, particularly in those living alone, additional social support may be needed. In Taiwan, the community living elderly maintained a higher cognitive function with social support. The lifestyle management program in Taiwan could promote better quality of life in individuals who lived alone (Yeh & Liu, 2003).

Higher levels of income were associated with a higher cognitive function in a Canadian cohort study (St John, Seary, Menec, & Tyas, 2016). In our study, according to the health insurance type revealed a positive relationship with healthy cognition and a stable economic state.

Despite the enumerated procedures, there were limitations in our study. MMSE‐DS is useful tool for measuring cognitive function in a cohort study; however, it does not reflect the activities of daily living in the general population. We did not screen for these activities, which is key for diagnosing dementia. Second, the graded duration of education was just scored. We attempted to determine the cut‐off point of duration in public education. It is difficult to derive the optimal duration of public education. The data showed 6 years of public education in the drawn figure. Third, we did not have additional information of various living type or marital status. In this study, we only investigated living with other family members or living alone. Various aspects of living, such as marriage and family member composition of the generation, can affect the daily life. Fourth, there is a limitation pertaining to the economic status based on the government health insurance type. We used this information to predict the economic status. Additional information, including housing and income, could help provide a better analysis.

In this study, the epidemiology of cognitive impairment in the highly elderly population was identified, and associated factors for predicting this were revealed. We could identify associated factors such as low education, the elderly living alone, and economic strength. The factors could be controlled through social interest and effort. As the world is aging, we should be more interested in these efforts to be healthy aging.

## CONFLICT OF INTEREST

No conflicts of interest to report.

## AUTHOR CONTRIBUTIONS

Hyeyun Kim, Woong‐Sub Park: Study design, Data collection & Analysis and writing. Sujin Lee, Bon D. Ku: Data collection & Analysis and writing. Su Geun Ham: Data collection.
